# The haemostatic arsenal of the pediatric cardiac surgeon

**DOI:** 10.3389/fcvm.2023.1210564

**Published:** 2023-10-13

**Authors:** Alexander Reynolds, William Novick, Oleksandr Yachhnik, Andriy Plylypets, Massimo Griselli

**Affiliations:** ^1^ Swansea University Medical School, Wales, United Kingdom; ^2^ Global Surgery Institute, University of Tennessee Health Science Center, Memphis, TN, United States; ^3^ Novick Cardiac Alliance, Memphis, TN, United States; ^4^ Department of Paediatric Cardiac Surgery, St Nicholas Children's Hospital, L'viv, Ukraine; ^5^ Department of Cardiothoracic Surgery, Royal Brompton & Harefield Hospitals, London, United Kingdom

**Keywords:** humanitarian paediatric cardiac surgery, haemostasis, paediatric cardiac anaesthesia, blood donation and transfusion, global surgery, haemodilution, cardiopulmanory bypass

## Introduction

The practice of pediatric cardiac surgery involves a variation of manipulation, transection and transplantation of the Heart and great vessels. As such, the substantial risk of haemorrhage is ever-present in the peri- and post-operative period. As summarised by Jonas, there are five broad principles of haemostasis in paediatric cardiac surgery; appropriate plane dissection, minimally-tensile suturing technique, optimized cardiopulmonary bypass, surgical site packing and administration of blood products and factors (1). The evidence base for each of these principles is variable, with few articles commenting on the role of dissection, surgical approach, or minimally-tensile suture lines to incidence of haemorrhage and effective haemostasis, whilst the use of blood products, coagulation factors and other haemostatic drugs have been the subject of extensive investigation, including randomised controlled trials. In this short review, we will briefly summarise the evidence base and comment on how we may better understand effective haemostatic strategies through future research enquiries, and how these strategies may vary in the developed vs. humanitarian surgical setting.

### Surgical technique

It is the most logical of all of the declared principles, that surgical technique is the highest correlation to haemorrhage. Haemostasis does not start after the administration of protamine and blood products at the end of the procedure; it begins from the skin incision. To cut is to induce bleeding; to cut on the most avascular planes is to minimise bleeding; and to cut on the Heart or the great vessels is to guarantee excessive bleeding. Skilful and for-thought dissection of the tissues, therefore, is the first and most fundamental haemostatic technique. Generous cauterisation of the subcutaneous, sternal and raw tissues on the under surface of the chest wall must be employed to ensure minimal blood loss occurs from the initial dissection phase of the operation. This includes any blood vessels encountered on approach to the mediastinum. Establishing a meticulous control of bleeding from the start, with a gentle handling of tissues and a good combination of diathermy (without excessive use, which induces tissue necrosis and predisposes to sepsis).Reoperation complicates this strategy as extensive fibrotic lesions often obscure the raw areas, preventing the surgeon from completely identifying them and thus leaving them at risk of bleeding during sternal retraction and pericardiotomy. Excessive cauterisation of the mesenchyme in the case of reoperation is therefore best prophylaxis for intra- and post-operative bleeding (1).

Suture technique, specifically vascular anastomosis, has been likened to the block and tackle principle; rather than one pulley acting against a force (mass ×  gravity) to lift an object, multiple pullies can share the force of an object, and so reduce the minimum effort required to move said object. In a surgical context, the pulleys refer to the number of suture bites in a vessel, and minimum effort refers to the tension that both vessel ends are subjected to. The greater the number of suture bites made before introducing tension, the less tension subjected to each individual bite. Continuous suturing before applying tension during end-to-end anastomoses is therefore the least tense are therefore most haemostatic of suturing techniques (1).

The final operative aspect to consider is the human factor of fatigue. Long procedures that incur a challenging haemostasis are more demanding on the individual surgeon. One technique employed by one of the authors is to ask for help from another colleague. This approach has a two-fold advantage; it gives the primary surgeon a rest interval, simultaneously allowing the other surgeon to explore a new surgical field with fresh eyes.

### Cardiopulmonary bypass (CPB) strategies

Logically, the smaller the patient, the greater the haemodilution required for successful CPB. The smallest patients therefore have greater dilution of platelets and clotting factors before commencing the main operative phase. Priming of the CPB circuit bridges the gap between the circulatory volume and the CPB volume; and therefore, investigating the haemostatic effect of different priming solutions is useful. Rauf demonstrated that addition of albumin to the primer improves intraoperative platelet count, though blood loss was similar in both trial groups- suggesting coagulopathy was unlikely to be the primary cause of bleeding in this population (2). Albumin may therefore be useful in preventing platelet derangement or coagulopathy. Additionally, the use of retrograde autologous priming, whilst demonstrable in adults, is less clearly effective in paediatrics (3).

Anticoagulation is inevitable to prevent activation of the intrinsic clotting cascade, and heparin's rapid action, in conjunction with its easy protamine-induced reversal, ensures that this drug remains a permanent aspect of the CPB protocol. Evidence suggests however that, whilst previously, a weight-based approach was taken to heparin dosage, there may be benefit to individualised dosage protocols (4). The findings of this randomized control trial were considerable- biochemically, the processes of both thrombogenesis and fibrinolysis are not mutually exclusive in one instance, and the individualised dosage of heparin seemed to show simultaneous suppression of both processes intra-operatively. This dynamically prevents inappropriate clot formation, whilst the clots that are formed are not broken down prematurely. Beyond the obvious haemostatic benefit, the evidence for this mechanism results in a lower degree on consumptive coagulopathy, diminished blood loss and lower need for transfusion of haemoatological factors.

Dilution of pro-thrombotic compounds is the primary haemostatic challenge resulting from CPB. Hypofibrinogenemia will limit the rate of coagulation cascade activity, as less fibrin cross-meshes can be formed at the site of platelet-aggregation. Replenishing fibrinogen extrinsically has demonstrated significant haemostatic benefit (5); and substituting fibrinogen concentrate with cryoprecipitate has demonstrated non-inferiority in a mixture of prospective randomised studies (6, 7). However, population sizes were too small to give strong statistical power and may benefit from meta-analysis following further data publication. In the adult cardiac surgical patients, whether to use fibrinogen concentrate or cryoprecipitate as a first-line therapy for the treatment of acquired hypofibrinogenemia continues to be a subject of intense debate in the United States. Fibrinogen concentrate has many potential advantages including a rapid administration, the predictability of dose response, and a lower risk for viral transmission. However, fibrinogen concentrate lacks some factors which may reduce its haemostatic efficacy in some cases. To date, evidence supporting the routine or prophylactic use of fibrinogen concentrate in the adult cardiac surgical patients is not robust, and larger studies are needed to confirm its value compared to cryoprecipitate, which has been the gold standard for treating acquired hypofibrinogenemia for almost 50 years (8).

### Haematological monitoring & product administration

**Figure 1:**
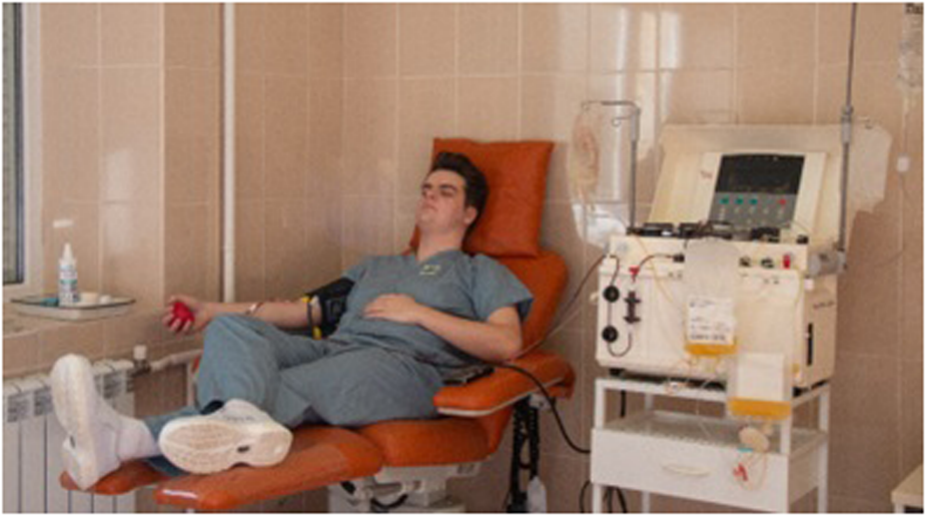
Pre-operative harvest of donor platelets from one author. Photographs taken by Pietro Chekal on behalf of Chernobyl Children International.

**Figure 2:**
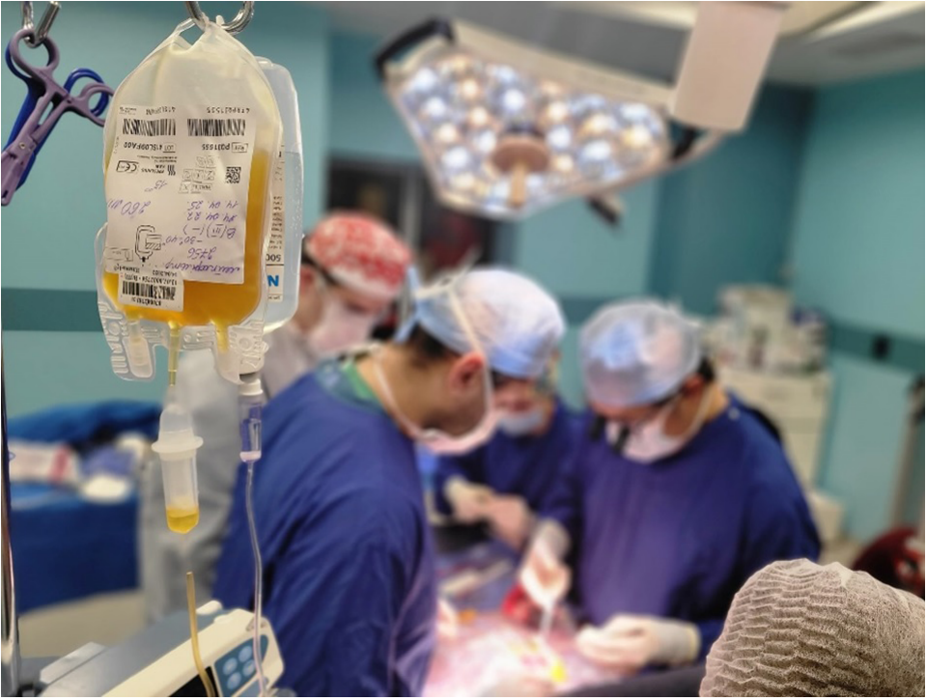
Intra-operative administration of the platelets from the author. Photographs taken by Pietro Chekal on behalf of Chernobyl Children International.

Surgical technique is the mainstay of haemostasis. However, intraoperative haematological monitoring should be performed. Fundamental attribution error is the psychological phenomenon of falsely believing a problem is due to one particular factor. Surgeons should be fully attentive to the surgical field and not allow deranged abnormal haematological tests to cause fundamental attribution error. The availability of coagulation tests in operating room should not distract the surgeon from performing a meticulous surgical haemostasis, ensuring there is not a “lack of prolene” instead of lack of coagulation factors. In the vast majority of cases, post-operative bleeding is a combination of heamatological and surgical deficit and their correction should be executed promptly by the surgical and anaesthetic teams,

Reacting to deranged haematology through clotting factor transfusion and appropriate protamine administration is key for the intraoperative medical management of the paediatric cardiac patient. It is the experience of the authors that, during humanitarian medical missions, blood product provision is not guaranteed. This can be due to a combination of factors, including a lack of blood transfusion infrastructure, lack of refrigeration for blood products, and large demand for blood in a 24-h period (especially in trauma facilities in conflict zones). In such circumstances where blood products are required immediately, members of the clinical team may consider donating their own blood. In our recent trip to Western Ukraine, one of the authors donated platelets (Figure 1) to a 2-year-old child who underwent correction of tetralogy of Fallot (Figure 2). Such donations should only be made when the donor is aware of their HIV status and has undergone laboratory follow-up from any needlestick injuries or other mechanisms through which blood-borne viruses may be transmitted. Where available, as was the case in this scenario, on-site testing of donor blood should be performed before donation. This minimised the risk to the patient, whilst offering the maximum benefit that allowed them to ultimately make a full recovery.

## Summary

The primary and secondary literature commented upon in this editorial indicates a multi-pronged approach is required to ensure minimal blood loss during pediatric cardiac surgery. Vascular-focussed operative skill, precise perfusion techniques, and anaesthetic intervention secondary to intraoperative haematological data are the fundamental principles of haemostasis in pediatric cardiac surgery, emphasising the role of interpersonal dynamics and multi-disciplinary collaboration for optimum results. In the humanitarian setting, it can be safe and feasible for team members to donate blood products when supply is scarce, and the clinical indication is clear. Patient selection is very important. Clinical prioritization of patients is essential when the availability of resources is critical. Patients who may require prolonged intensive care stay after surgery (often beyond the time our missions are completed) could be referred to cardiac units in the neighbouring countries with better facilities or planned at different time if clinical conditions allow. Again, patients who are likely to require some kind of mechanical circulatory support should be moved elsewhere if possible. Surgical lists during these missions should be rationalized in accordance with the resources available (sutures, patches, valves, conduits, drains, etc) without compromising the safety and success of the procedures. Whilst the diversity in haemostatic variables is clear, the common factor in all of them is their need for further investigation through randomised controlled trials and subsequent meta-analysis.
